# Wounding and UVB Light Synergistically Induce the Biosynthesis of Phenolic Compounds and Ascorbic Acid in Red Prickly Pears (*Opuntia ficus-indica* cv. Rojo Vigor)

**DOI:** 10.3390/ijms20215327

**Published:** 2019-10-25

**Authors:** Erika Ortega-Hernández, Vimal Nair, Jorge Welti-Chanes, Luis Cisneros-Zevallos, Daniel A. Jacobo-Velázquez

**Affiliations:** 1 Tecnologico de Monterrey, Escuela de Ingenieria y Ciencias, Av. General Ramon Corona 2514, Colonia Nuevo Mexico, Zapopan 45138, Jal., Mexico; erika.orhe@gmail.com; 2 Department of Horticultural Sciences, Texas A&M University, College Station, TX 77843-2133, USA; vimal.nair16@gmail.com (V.N.); lcisnero@tamu.edu (L.C.-Z.); 3 Tecnologico de Monterrey, Escuela de Ingenieria y Ciencias, Av. Eugenio Garza Sada 2501 Sur, Colonia Tecnologico, Monterrey, NL 64849, Mexico; jwelti@tec.mx

**Keywords:** UVB light, wounding, phenolic compounds, ascorbic acid, l-galactono-γ-lactone dehydrogenase (GalLDH), phenylalanine ammonia-lyase (PAL), prickly pear

## Abstract

The present study evaluated the effects of ultraviolet B (UVB) radiation and wounding stress, applied alone or combined, on the biosynthesis of phenolic compounds and ascorbic acid in the peel and pulp of red prickly pear (*Opuntia ficus-indica* cv. Rojo Vigor). Whole and wounded-fruit samples were treated with UVB radiation (6.4 W·m^−2^) for 0 and 15 min, and stored for 24 h at 16 °C. Phytochemical analyses were performed separately in the peel and pulp. The highest phenolic accumulation occurred after storage of the whole tissue treated with UVB, where the main phenolic compounds accumulated in the peel and pulp were quercetin, sinapic acid, kaempferol, rosmarinic acid, and sinapoyl malate, showing increases of 709.8%, 570.2%, 442.8%, 439.9%, and 186.2%, respectively, as compared with the control before storage. Phenylalanine ammonia-lyase (PAL) activity was increased after storage of the whole and wounded tissue treated with UVB light, and this increase in PAL activity was associated to phenolic accumulation. On the other hand, l-galactono-γ-lactone dehydrogenase (GalLDH) activity and ascorbic acid biosynthesis was enhanced due to UVB radiation, and the effect was increased when UVB was applied in the wounded tissue showing 125.1% and 94.1% higher vitamin C content after storage when compared with the control. Respiration rate was increased due to wounding stress, whereas ethylene production was increased by wounding and UVB radiation in prickly pears. Results allowed the generation of a physiological model explaining the UVB and wound-induced accumulation of phenolic compounds and ascorbic acid in prickly pears, where wounding facilitates UVB to access the underlying tissue and enhances an apparent synergistic response.

## 1. Introduction

*Opuntia ficus-indica* (L.) Mill., commonly known as prickly pear, is a tropical or subtropical plant that belongs to the Cactaceae family and is mainly used for fruit production [1]. It has been widely used in Mexico for the treatment of different health disorders including ulcers, cancer, and high levels of cholesterol [2]. Its biological properties have been attributed to different phytochemicals such as flavonoids and ascorbic acid. The beneficial characteristics of these bioactive molecules are based on the regulation of metabolic processes in the organism trough neutralization of free radicals, activation of enzymes, inhibition of cell receptors, and modulation of gene expression [3].

Phenolic compounds are secondary metabolites produced via the shikimate and phenylpropanoid pathways [4]. Their presence has been detected in cactus fruit both in peel and pulp [5]. It has been found that their antioxidant activity is mainly due to the major flavonoids present in the cactus pear fruit—quercetin, kaempferol, and isorhamnetin [6]. On the other hand, ascorbic acid is a water-soluble vitamin that plays beneficial roles in preserving bones, generating blood vessels, and maintaining healthy skin. Prickly pear fruits exhibit an ascorbic acid content of 20 to 40 mg/100 g fresh weight [7], such content is higher than that found in apples, pears, grapes, and bananas [8].

In the past years, abiotic stresses such as wounding and ultraviolet (UV) radiation have been reported to be an effective tool that induces the accumulation of bioactive compounds in plants [9]. This stress-response is mediated through an immediate production of stress-signaling molecules that activate the expression of primary and secondary metabolism related genes, which are latter translated into enzymes involved in the biosynthesis of secondary metabolites [10].

The use of plants for the production and isolation of secondary metabolites can be promoted by ultraviolet B (UVB) radiation [11]. UVB radiation (280–315 nm) is a key signal that induces the necessary processes of photosynthetic recovery and the mechanisms of photoprotection for the physicochemical acclimatization and survival of plants [12]. Plants accumulate in different cell compartments, preferably in cells of the epidermis, phenolic compounds, capable of attenuating UV spectrum without interfering with the absorption of photosynthetic active radiation [13].

The objective of this study was to determine the effect of UVB radiation and wounding stress on the accumulation of phenolic compounds and ascorbic acid in the peel and pulp of red prickly pear (*Opuntia ficus-indica* cv. Rojo vigor), and the role of enzymes involved in their biosynthesis including phenylalanine ammonia-lyase (PAL) and l-galactono-γ-lactone dehydrogenase (GalLDH), as well as the respiration rate and production of volatile organic compounds (VOCs) associated to ethylene.

## 2. Results and Discussion

### 2.1. Phenolic Profile of Red Prickly Pear Pulp and Peel

**Figure 1:**
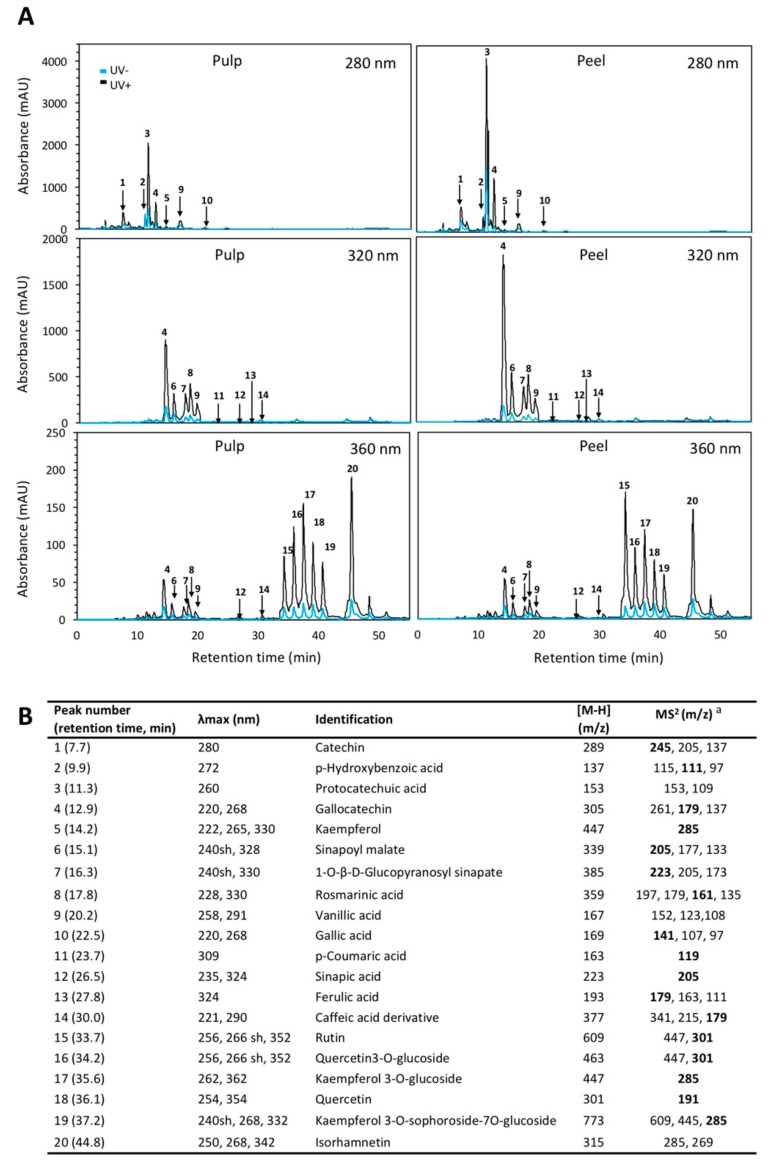
(**A**) Typical HPLC-diode array detector (DAD) chromatogram, shown at 280, 320, and 360 nm of identified phenolic compounds from methanol/water (50:50, *v*/*v*) extracts obtained from red prickly pears pulp and peel treated and non-treated with ultraviolet B (UVB) light for 15 min and stored for 24 h at 16 °C. (**B**) Identification of individual phenolic compounds in red prickly pear. Identification was obtained by HPLC-DAD and HPLC-electrospray ionization (ESI)–sequential mass spectrometry (MS^n^). Abbreviations: shoulder (sh). ^a^ Major fragment ions are highlighted in bold.

Results from the HPLC-diode array detector (DAD)-sequential mass spectrometry (MS^n^) phenolic profiles of red prickly pear is shown in Figure 1. The major compound detected in the pulp was protocatechuic acid followed by *p*-hydroxybenzoic acid and kaempferol in pulp (Table 1), whereas sinapoyl malate and quercetin were the major phenolic compounds in the peel (Table 2).

The phenolic profile of red prickly pear obtained herein is similar to those previously reported for five different varieties of prickly pear [5,14]. However, Abdel-Hameed et al. [14] reported gallic acid and rutin as the major compounds in the peel and pulp (22.34–43.74 mg/100 g dry weight (DW)) of red cultivars, whereas Yeddes et al. [5] reported isorhamnetin (45.59 ± 0.01 mg/100 g DW) as the major phenolic in the peel. Differences in the phenolic profile reported herein and previous reports could be attributed to multiple factors, including plant variety, maturity stage, growing conditions, and method of analysis [4].

### 2.2. Immediate Effect of UVB Radiation on the Phenolic Profile of Whole and Wounded Red Prickly Pear Pulp

The application of UVB induced an immediate significant change in the content of certain phenolic compounds present in the pulp when the whole fruit was irradiated (Table 1). For instance, the fruit pulp of whole red prickly pear irradiated showed significant increment in protocatechuic acid (31.8%), kaempferol (27.1%), 1-*O*-β-d-glucopyranosyl sinapate (38.8%), rutin (14.2%), kaempferol 3-*O*-glucoside (58.3%), and kaempferol 3-*O*-sophoroside-7*O*-glucoside (45.7%) as compared to non-irradiated samples, whereas UVB radiation induced slight decrements in catechin (−11.6%) and gallocatechin (−17.1%).

Furthermore, UVB-irradiated wounded pulp showed an increase in kaempferol (33.81%), sinapoyl malate (59.3%), 1-*O*-β-d-glucopyranosyl sinapate (58.9%), rosmarinic acid (48.2%), vanillic acid (14.4%), *p*-coumaric acid (440.4%), sinapic acid (212.5%), ferulic acid (393.3%), caffeic acid derivative (475.9%), quercetin 3-*O*-glucoside (367.4%), kaempferol 3-*O*-glucoside (177.7%), quercetin (43.8%), and kaempferol 3-*O*-sophoroside-7*O*-glucoside (87.2%) as compared with the pulp of non-irradiated whole fruits (Table 1). On the other hand, the wounded pulp irradiated with UVB showed lower levels of catechin (−29.4%) and gallocatechin (−26.2%) after UVB radiation. 

### 2.3. Effect of Storage Time on the Phenolic Profile of Whole and Wounded Red Prickly Pear Pulp Treated with UVB Radiation 

After 24 h of storage, there was a slight increment in the content of rutin (15.4%) and a decrease of rosmarinic acid (−22.9%) and kaempferol 3-*O*-sophoroside-7*O*-glucoside (−13.2%) in the pulp of untreated whole red prickly pear (Table 1). On the other hand, wounding stress alone induced a significant increase in the content of *p*-coumaric acid (26.4%), sinapic acid (54.6%), ferulic acid (21.2%), caffeic acid derivative (38.3%), quercetin 3-*O*-glucoside (146.7%), and kaempferol 3-*O*-glucoside (30.1%) in the pulp.

Moreover, the content of phenolic compounds significantly increased after 24 h of storage in most samples treated with UVB light alone or combined with wounding stress. In the whole fruit pulp treated with UVB, there was a significant increment in protocatechuic acid (52.2%), kaempferol (45.1%), sinapoyl malate (160.7%), 1-*O*-β-d-glucopyranosyl sinapate (62.1%), rosmarinic acid (427.8%), sinapic acid (33.3%), rutin (14.3%), quercetin 3-*O*-glucoside (36.9%), quercetin (33.1%), and kaempferol 3-*O*-sophoroside-7*O*-glucoside (36.8%) as compared with the untreated control. On the other hand, the wounded fruit pulp treated with UVB light showed a significant increment in the content of kaempferol (167.9%), sinapoyl malate (247.9%), 1-*O*-β-d-glucopyranosyl sinapate (572.3%), rosmarinic acid (360.3%), sinapic acid (21.1%), kaempferol 3-*O*-glucoside (1704.3%), kaempferol 3-*O*-sophoroside-7*O*-glucoside (106.2%), quercetin 3-*O*-glucoside (759.6%), and quercetin (57.1%) as compared with the untreated control. Furthermore, a decrement in catechin (−47.3%), ferulic acid (−10.1%), and *p*-coumaric acid (−38.9%) was detected. 

### 2.4. Immediate Effect of UVB Radiation on the Phenolic Profile of Whole and Wounded Red Prickly Pear Peel

As shown in Table 2, after UVB treatment, the peel of the whole fruit showed an immediate increment in kaempferol (535.7%), sinapoyl malate (56.6%), 1-*O*-β-d-glucopyranosyl sinapate (280.8%), rosmarinic acid (124.9%), *p-*coumaric acid (45.0%), sinapic acid (147.1%), quercetin 3-*O*-glucoside (143.6%), kaempferol 3-*O*-glucoside (123.6%), quercetin (257.3%), and kaempferol 3-*O*-sophoroside-7*O*-glucoside (266.5%) as compared with the untreated control, whereas a decrease in *p*-hydroxybenzoic acid (−46.4%), protocatechuic acid (−52.2%), gallocatechin (−17.9%), and vanillic acid (−12.8%) content was detected.

Furthermore, UVB radiation applied in the wounded fruit induced an immediate increase in the content of kaempferol (377.1%), sinapoyl malate (132.5%), 1-*O*-β-d-glucopyranosyl sinapate (334.5%), rosmarinic acid (266.6%), *p*-coumaric acid (534.1%), sinapic acid (516.1%), ferulic acid (1264.3%), caffeic acid derivative (152.7%), quercetin 3-*O*-glucoside (78.4%), kaempferol 3-*O*-glucoside (38.2%), quercetin (341.3%), and kaempferol 3-*O*-sophoroside-7*O*-glucoside (81.7%) in the peel of the fruit as compared with the untreated control (Table 2). Interestingly, UVB radiation applied in wounded-tissue decreased the UVB-induced immediate accumulation of catechin (-18.8%), *p*-hydroxybenzoic acid (−52.7%), protocatechuic acid (−14.1%), gallocatechin (−27.6%), and vanillic acid (−14.7%). 

### 2.5. Effect of Storage Time on the Phenolic Profile of Whole and Wounded Red Prickly Pear Peel Treated with UVB Radiation

After storage, the peel of untreated stored whole fruit showed a significant increment in the content of sinapoyl malate (35.3%), 1-*O*-β-d-glucopyranosyl sinapate (153.4%), rosmarinic acid (40.8%), *p*-coumaric acid (37.9%), sinapic acid (91.8%), ferulic acid (88.7%), and caffeic acid derivative (265.6%), and a decrease of *p*-hydroxybenzoic acid (−17.9%), protocatechuic acid (−13.8%), and rutin (−19.2%), whereas the wounded peel showed increases in the content of kaempferol 3-*O*-sophoroside-7*O*-glucoside (49.2%) as compared with the peel from stored whole fruits. As earlier described, phenolic compounds accumulation during storage is triggered by oxidative stress, and their biosynthesis is associated with the wound-healing process to prevent water loss during storage of wounded-tissue [15]. Furthermore, other genes (i.e., chalcone synthase) involved in the biosynthesis of flavonoids such as kaempferol and quercetin have also been reported to be activated by the application of wounding stress in plants [10].

Moreover, the content of phenolic compounds significantly increased after 24 h of storage in most samples treated with UVB light alone or combined with wounding stress. The whole peel treated with UVB light showed an increment in the content of kaempferol (442.8%), sinapoyl malate (186.2%), 1-*O*-β-d-glucopyranosyl sinapate (391.8%), rosmarinic acid (439.9%), sinapic acid (570.2%), ferulic acid (245.9%), quercetin 3-*O*-glucoside (117.7%), kaempferol 3-*O*-glucoside (139.9%), quercetin (709.8%), and kaempferol 3-*O*-sophoroside-7*O*-glucoside (293.3%) as compared with the untreated control. Likewise, the peel of whole fruits treated with UVB light showed decrements in the content of *p-*hydroxybenzoic acid (-70.7%), gallocatechin (−36.9%), and vanillic acid (−51.5%).

On the other hand, after storage of wounded red prickly pear treated with UVB light, the peel showed a significant increment in kaempferol (288.1%), sinapoyl malate (264.5%), 1-*O*-β-d-glucopyranosyl sinapate (454.4%), rosmarinic acid (302.1%), *p*-coumaric acid (114.9%), sinapic acid (967.3%), ferulic acid (258.8%), caffeic acid derivative (304.9%), quercetin 3-*O*-glucoside (72.6%), kaempferol 3-*O*-glucoside (63.8%), and quercetin (505.9%) as compared with non-treated whole peels. On the other hand, decrements were detected in catechin (−30.8%), *p-*hydroxybenzoic acid (−80.8%), protocatechuic acid (−28.9%), gallocatechin (−59.9%), vanillic acid (−31.4%), rutin (−27.8%), and isorhamnetin (−41.9%) after storage of UVB treated wounded peel samples.

Flavonoids that are induced by UVB may have functions to reduce the effects of UVB damage, which include both reactive oxygen species (ROS) scavenging and UVB screening properties. In the present study, the increase of sinapoyl malate content in UVB-treated peels agrees with results from previous experiments performed in *Arabidopsis thaliana*, *Brassica napus*, and *Brassica oleracea* where UVB radiation induced the accumulation of sinapoyl malate and other sinapates that strongly absorb in the UV range, protecting the plant against UVB radiation [16,17,18,19]. Furthermore, the accumulation of specific flavonoid glycosides appears to be an intrinsic part of UVB response, with the gene expression of uridine 5’-diphospho (UDP)-glucuronosyltransferase being directly controlled by UVB radiation. The increase in concentration of kaempferol and its glycoside in UVB-treated peels agrees with previous reports performed in *Brassica rapa* ssp. *Chinensis* [20].

In addition to the accumulation of kaempferol and sinapates observed in the pulp and peel, increases in rosmarinic acid, *p*-coumaric acid, quercetin, and its derivatives were detected in the peel immediately after UVB treatment. The synthesis of rosmarinic acid has its initial formation from tyrosine and then binds in the phenylpropanoid pathway with a *p*-coumaric acid derivative. According to previous literature, UVB radiation increases the availability of tyrosine [21], thus increasing its concentration as substrate for rosmarinic acid biosynthesis. On the other hand, it has been reported that quercetin and kaempferol have similar extinction coefficients in the UV region of the spectrum and are produced in the greatest quantities as a response to UVB radiation in various edible species [22,23] because they accumulate directly in the vacuoles of epidermal and subepidermal cell layers [24,25]. Furthermore, quercetin is a more efficient ROS scavenger than kaempferol due to its higher number of hydroxyl groups. This characteristic is important and could explain the difference in the profile of both tissues, as in the whole red prickly pear fruit under irradiation, the peel is the only protection barrier that directly receives all the radiation. In addition to the immediate decrease in gallocatechin and catechin in the pulp and peel, the decrease in *p-*hydroxybenzoic acid and protocatechuic acid can also be explained in terms of carbon flux, as there is a deviation of carbon skeletons towards the biosynthesis of rosmarinic acid from *p*-coumaric acid, which is the precursor of these phenolic compounds.

Likewise, immediate increases in the content of hydroxycinnamic acids (i.e., *p*-coumaric acid, sinapic acid, ferulic acid, and caffeic acid derivative) were observed as a response to wounding stress. The increase in phenolic compounds was higher when UVB was applied in the wounded pulp as compared with the whole fruit, showing an immediate synergistic effect between wounding and UVB radiation. These results agree with a previous report, where the application of UVB radiation (for 15 and 90 min) in wounded prickly pear induced an immediate increase in the total phenolic content of the pulp [26]. The accumulation of hydroxycinnamic acids in wounded tissue treated with UVB radiation can be attributed to their participation in lignification during the wound-healing process [15] and UVB protection [27]. These results agree with previous reports were wounded carrots treated with UVB showed accumulation of hydroxycinnamic acids, mainly chlorogenic acid, ferulic acid, and 3,5-dicaffeoylquinic acid [28]. Likewise, the higher accumulation of phenolic compounds detected in the irradiated wounded tissue as compared with the control could be attributed to the higher area of exposure of the tissue to UVB radiation, as in the case of the whole fruit the pulp was partially protected by the peel, which contains compounds that filter UVB light.

Furthermore, after storage, results showed an increase in the glycosylation of the flavonoids after UVB radiation. This effect can be related to an up-regulation of genes homologous to the UDP-glycosyltransferase family protein, UGT73B2, which catalyzes the glycosylation of flavonoids from UDP-glucose. Mewis et al. [29] have reported previously a 3.5-fold induction of such genes 24 h after the UVB treatment of broccoli sprouts. Moreover, UVB could also be stimulating the production of nitric oxide (NO), which may up-regulate the expression of transcription factor ELONGATED HYPOCOTYL5 (HY5), and its final target genes such as chalcone synthase (CHS) [30], inducing the accumulation of flavonoids and derivatives to absorb UVB and also to scavenge ROS produced by wounding in combination with UVB radiation stress [31].

In contrast with the observation as an immediate response to UVB radiation, after 24 h of storage the application of wounding stress decreased the UVB-induced accumulation of individual phenolic compounds in the peel. These results are in agreement with a previous report where the content of total phenolic compounds was evaluated after storage of prickly pear treated under the same conditions herein evaluated [26]. This phenomenon could be explained in terms of the balance between biosynthesis and utilization rate of phenolic compounds for the synthesis of lignin. It is likely that after 24 h of storage, the conversion rate of hydroxycinnamic acids into monolignols was higher than their biosynthesis rate in prickly pear treated with wounding stress and UVB [32].

### 2.6. Phenylalanine Ammonia-Lyase (PAL) Activity of Whole and Wounded Red Prickly Pear Treated with UVB Radiation

**Figure 2:**
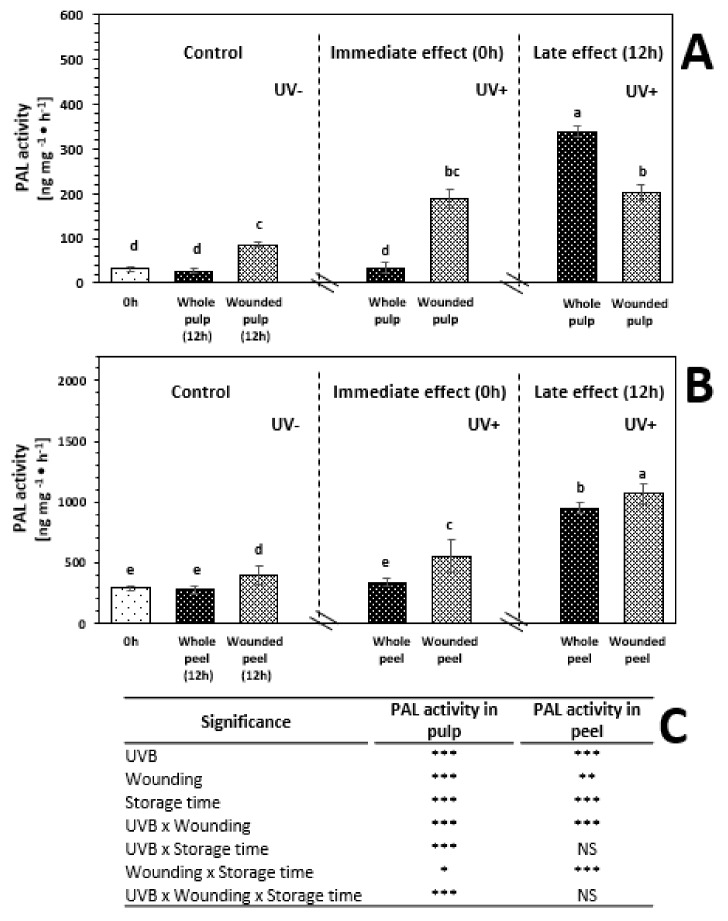
Enzymatic activity of phenylalanine ammonia-lyase (PAL) before and after storage (12 h at 16 °C) of whole and wounded red prickly pear pulp (**A**) and peel (**B**) treated (UV+) and non-treated (UV−) with UVB light (6.4 W·m^−2^) for 15 min. (**C**) Full factorial analyses of variance showing the main effects and interactions of the variables evaluated. Bars are means of four replicates ± standard error of the mean. Different letters among bars indicate statistical difference between treatments using the least significant difference (LSD) test (*p* < 0.05). Asterisks indicate that main effects and interactions are significantly different by analyses of variance (ANOVA). NS—non significant, * *p* < 0.05, ** *p* < 0.01, *** *p* < 0.001. Results are expressed as ng of trans-cinnamic acid produced per mg of protein per hour.

The enzymatic activity of PAL before and after 12 h of storage of red prickly pear treated and non-treated with UVB is shown in Figure 2. As observed, the enzymatic activity of PAL was 841.2% higher in the peel than in the pulp, which is consistent with the higher content of phenolic compounds detected in the peel (Table 1 and Table 2).

The application of UVB treatment did not induce an immediate significant increment in PAL activity of the pulp and peel when the whole fruit was treated with UVB (Figure 2). However, when the wounded fruit was subjected to UVB, there was a significant increase on the enzymatic activity of PAL of 505.1% and 88.4% in the wounded pulp and peel, respectively, as compared with the untreated control. The immediate increment in PAL activity in wounded tissue treated with UVB corresponded to an increment in the content of phenolic compounds (Table 1). These results confirmed that as an immediate response to the combination of both stresses, the biosynthesis of phenolic compounds was elicited.

In addition, PAL activity was evaluated at 12 h of storage to determine if the phenylpropanoid metabolism was further enhanced as a late response to wounding and UVB stresses. There was no significant increment in the enzymatic activity of PAL of untreated whole red prickly pear fruit pulp and peel after 12 h of storage. On the other hand, wounding stress alone induced a significant increment in the PAL activity of the pulp (173.1%) and peel (36.1%). This increment in PAL activity can be related to production of ROS and ethylene due to wounding stress during storage [10,33].

The application of UVB light alone in the whole pulp and peel induced an increment in the enzymatic activity of PAL after 12 h of storage; these samples showed a significant increase of 983.7% and 222.3%, respectively, as compared with the untreated control. On the other hand, the application of UVB light did not increase the wound-induced accumulation of phenolic compounds in the pulp, whereas the peel showed 263.1% higher PAL activity as compared with the untreated control. These results suggest that the application of wounding stress and UVB light induce an immediate response of PAL which peaks earlier and decreases after 12h compared to wounding alone. This response would generate earlier biosynthesis of phenolic compounds and its utilization (Table 1).

### 2.7. Effect of UVB, Wounding Stress, and Storage Time on Total Ascorbic Acid Content of Red Prickly Pear

**Figure 3:**
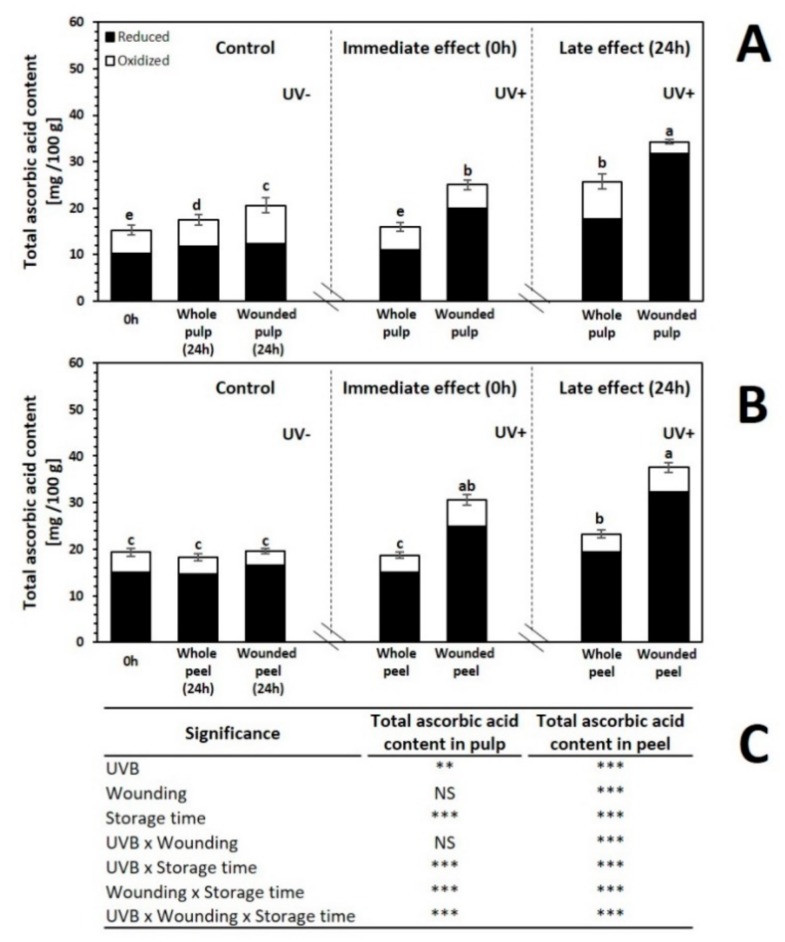
Concentration of ascorbic acid before and after storage (24 h at 16 °C) of whole and wounded red prickly pear (**A**) pulp and (**B**) peel treated with UVB light (6.4 W·m^−2^) for 0 and 15 min. (**C**) Full factorial analyses of variance showing the main effects and interactions of the variables evaluated. Bars are means of five replicates ± standard error. Different letters among bars indicate statistical difference between treatments using the least significant difference (LSD) test (*p* < 0.05). Asterisks indicate that main effects and interactions are significantly different by analyses of variance (ANOVA). NS—non significant, * *p* < 0.05, ** *p* < 0.01, *** *p* < 0.001. Results are expressed as mg equivalents of ascorbic acid per 100 g of sample in dry weight basis.

The ascorbic acid content before and after 24 h of storage of red prickly pear exposed to different UVB light doses (6.4 W·m^−2^ for 0 and 15 min) is shown in Figure 3. As observed, the content of ascorbic acid is similar in the pulp and the peel. Similar values of ascorbic acid content have been previously reported in different species of prickly pear [34].

The whole pulp and peel did not show immediate significant changes with the application of UVB treatment. On the other hand, the wounded pulp treated with UVB showed an increment in the content of ascorbic acid of 64.1% as compared to the control, whereas the wounded peel presented significant increments of 58.4%. This immediate increase in ascorbic acid levels could be related with a defense mechanism of the plant activated after UV radiation of the exposed underlying tissue after peel removal [35].

After storage, the application of UVB alone induced a significant increment in ascorbic acid content in the whole fruit pulp (46.7%). Furthermore, after storage, the ascorbic acid content in the peel of whole fruits treated with UVB increased by 27.7%, as compared with the untreated sample stored for 24 h. Castagna et al. [36] obtained similar results; the authors reported an increase in total ascorbic acid concentration of 34–41% for the flesh of tomato after 10 d of storage of fruits treated for 1 h of UVB (1.69 W·/m^2^) as compared with the control. As earlier mentioned, UV-radiation produces ROS in the plant tissue, and, thus, ascorbic acid biosynthesis during storage would be acting as an antioxidant or would be regenerating antioxidant enzymes to neutralize free radicals.

When the two stresses were combined (wounding + UVB light), a significant increase of 76.8% in the concentration of ascorbic acid after storage of wounded pulp treated with 15 min was induced as compared with the wounded control stored for 24 h. Likewise, the wounded peel treated with 15 min of UVB light showed a significant increase of ascorbic acid of 94.2%, as compared with the stored wounded control. In previous reports, non-significant changes in ascorbic acid content due to wounding stress was found in different plants including sweet potato, radish, and red cabbage [37]. However, the combined effect of wounding stress and UVB on ascorbic acid had shown a significant increase of 67.2% in the pulp and 84.6% in the peel of prickly pear after 24 h of storage [26]. UVB and wounding stress presented a synergistic effect on the elicitation of ascorbic acid. This apparent synergistic effect could be attributed to the removal of the peel, which would allow UVB to access the underlying tissue which would respond to the UVB stress. Thus, UVB induces the monomerization and accumulation of UV RESISTANCE LOCUS 8 photoreceptor (UVR8) photoreceptor, which initiates a sequence of metabolic events leading with the expression of genes related with the synthesis of secondary metabolites, including vitamin C [38]; likewise UVB induces the generation of ROS, enhancing the wound-induced production of ROS, which also is an important signaling molecule involved in the production of antioxidants in plants [10,28,33].

### 2.8. l-Galactono-γ-Lactone Dehydrogenase (GalLDH) Activity

**Figure 4:**
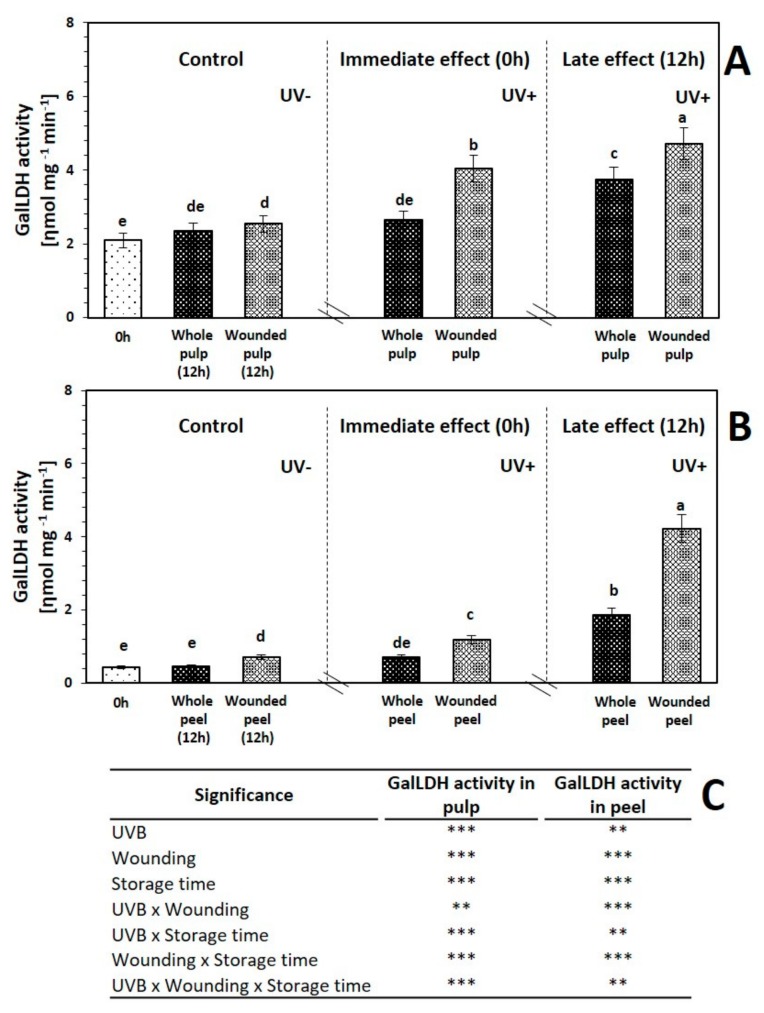
Enzymatic activit y of l-galactono-γ-lactone dehydrogenase (GalLDH) before and after storage (12 h at 16 °C) of whole and wounded red prickly pear (**A**) pulp and (**B**) peel treated with UVB light (6.4 W·m^−2^) for 0 and 15 min. (**C**) Full factorial analyses of variance showing the main effects and interactions of the variables evaluated. Bars are means of three replicates ± standard error. Different letters among bars indicate statistical difference between treatments using the least significant difference (LSD) test (*p* < 0.05). Asterisks indicate that main effects and interactions are significantly different by analyses of variance (ANOVA). NS—non significant, * *p* < 0.05, ** *p* < 0.01, *** *p* < 0.001. Results are expressed as oxidized ηmol L-Gal per mg of protein per minute.

GalLDH is a key enzyme in the l-galactose pathway that results on the production of vitamin C. This enzyme catalyzes the production of L-galactose lactone, the ascorbic acid precursor. The enzymatic activity of GalLDH before and after 12 h of storage of red prickly pear subjected to wounding and UVB is shown in Figure 4. As observed, the pulp showed 377.57% higher enzymatic activity of GalLDH than peel, whereas the concentration of ascorbic acid was similar in both tissues.

The application of UVB treatment did not induce an immediate significant increment in the enzymatic activity of GalLDH of whole red prickly pear fruit pulp and peel. On the other hand, wounding stress combined with UVB treatment induced an increment in the enzymatic activity of GalLDH in the pulp (93.4%) and peel (172.49%) as compared with the control. GalLDH activity presented the same trend with the accumulation of ascorbic acid in response to the wounding stress. After 12 h of storage, no significant changes in GalLDH activity was detected in untreated whole red prickly pear fruit tissues.

The application of UVB light alone or in combination with wounding stress induced an increment in the enzymatic activity of GalLDH after 12 h of storage. Regarding the whole fruit pulp and peel treated with UVB radiation, these samples showed a significant increase of 78.7% and 328.9%, respectively, as compared with the untreated control after 12 h of storage, whereas the wounded tissues treated with UVB light showed a significant increase of GalLDH of 125.6% in the pulp and 865.8% in the peel, as compared with the untreated control. The results suggest that the application of wounding stress and UVB light have a synergistic effect in the GalLDH activity.

GalLDH activity presented the same trend and a positive correlation (*R^2^* = 87%) with the content of ascorbic acid. These results illustrated that the changes in GalLDH enzyme activities influenced the changes in ascorbic acid content, increasing mainly as an immediate and late response to wounding stress in combination with UVB light. The results were consistent with those obtained from tomato leaf [38], mung bean sprouts [39], and lettuce [40]. Furthermore, the results of induction of GalLDH activity and ascorbic acid production may be mediated through UVB-induced transcription factors, which are enhanced once the peel is removed, allowing UVB receptors to be triggered. Furthermore, ascorbic acid produced when UVB is applied alone or combined with wounding stress could be used to ameliorate the stress-induced production of ROS and to support ethylene production, as shown in the following section [41].

### 2.9. Respiration Rate and Ethylene Analysis

**Figure 5:**
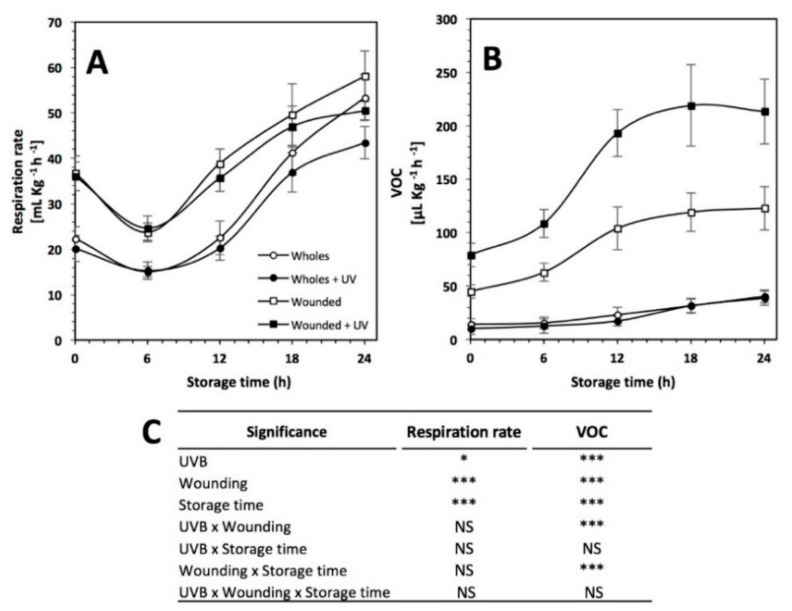
(**A**) Respiration rate and (**B**) volatile organic compounds (VOCs) production in untreated red prickly pear, treated with UVB light (6.4 W·m^−2^) for 15 min alone, treated with wounding stress alone, and treated with both UVB light and wounding stress during storage time (0, 6, 12, 18, and 24 h) at 16 °C. (**C**) Full factorial analyses of variance showing the main effects and interactions of the variables evaluated. Values represent the mean of five replicates with their standard error bars. Asterisks indicate that main effects and interactions are significantly different by analyses of variance (ANOVA). NS—non significant, * *p* < 0.05, ** *p* < 0.01, *** *p* < 0.001.

The respiration rate and VOCs production (related with ethylene) before and during storage (0 to 24 h) of red prickly pear treated and non-treated with wounding and/or UVB light is shown in Figure 5. Prickly pear is a non-climacteric fruit having a low respiration rate (22.34 mL CO_2_ × Kg^−1^ × h^−1^) and ethylene production (10.27 μL C_2_H_4_ × Kg^−1^ × h^−1^) at 20 °C. Similar values have been previously reported by Corrales-García et al. [42] for Burrona and Cristalina prickly pear varieties stored at room temperature.

Respiration rate was significantly affected by UVB light exposure (*p* < 0.05), storage time and by wounding stress (*p* < 0.001) (Figure 5A). Immediately after wounding stress, the respiration rate of red prickly pear increased by 61.4%. During storage, the respiration rate of whole samples was lower than wounded samples, whereas no significant difference was detected between irradiated and non-irradiated samples. At 6 h of storage, samples showed a decrease in the respiration rate. Thereafter, respiration rate increased relatively constant in all samples until the end of storage time. The highest respiration rate was observed with the treatment of wounding stress alone at 24 h, being 12.1% higher than the non-treated samples. Wound-induced respiration has been related to an enhanced synthesis of enzymes involved in the respiration pathway, such as ATP-dependent phosphofructokinase involved in carbohydrate breakdown leading to pyruvate [43] and to an enhanced aerobic mitochondrial respiration related to changes in mitochondrial structure [44]. Wound-induced respiration may also be associated in part to α-oxidation of long-chain fatty acids from membrane deteriorative processes [45]. On the other hand, there is scarce information on the effect of UVB on cell respiration. It has been reported that an increase in cell respiration is related to the greater need for energy to protect against UVB and for repair of UVB damage [46]. However, the reason for the inhibitory effect of UVB light on respiration has not been elucidated. It can be attributed to damage of mitochondrial structures that might result in decreased respiration.

Ethylene was indirectly measured in this study by determining VOCs (Figure 5B). UVB light, wounding stress, storage time, and the interaction between UVB light with wounding and wounding with storage time showed a significant effect on VOCs production (*p* < 0.001). The application of wounding stress alone and the combination of UVB with wounding stress showed an immediate increase of 337.4% and 670.4%, respectively, in VOCs production, whereas the UVB treatment alone did not significantly affect VOC production in red prickly pear as an immediate response. Aside from this, the prickly pear showed a constant increase in the ethylene production until the end of storage time in the samples treated with wounding stress alone (122.78 mL VOCs Kg^−1^h^−1^) and wounding stress combined with UVB light (213.34 mL VOCs Kg^−1^h^−1^), being 216.85% and 450.6% higher than the control, respectively. The stress-induced production of ethylene and increases in respiration rate have been observed in various plant species such as melon [47], *Arabidopsis* [48], broccoli [49], and carrot [10,50]. These results suggest an apparent synergistic effect between wounding stress and UVB light in the production of ethylene, where wounding allows UVB to act on the underlying tissue. The application of UVB alone did not have any significant change in ethylene production. However, when combined with wounding, production increased twofold.

### 2.10. Potential Mechanism for the UVB and Wound-Induced Accumulation of Phenolic Compounds and Ascorbic Acid in Prickly Pear

**Figure 6:**
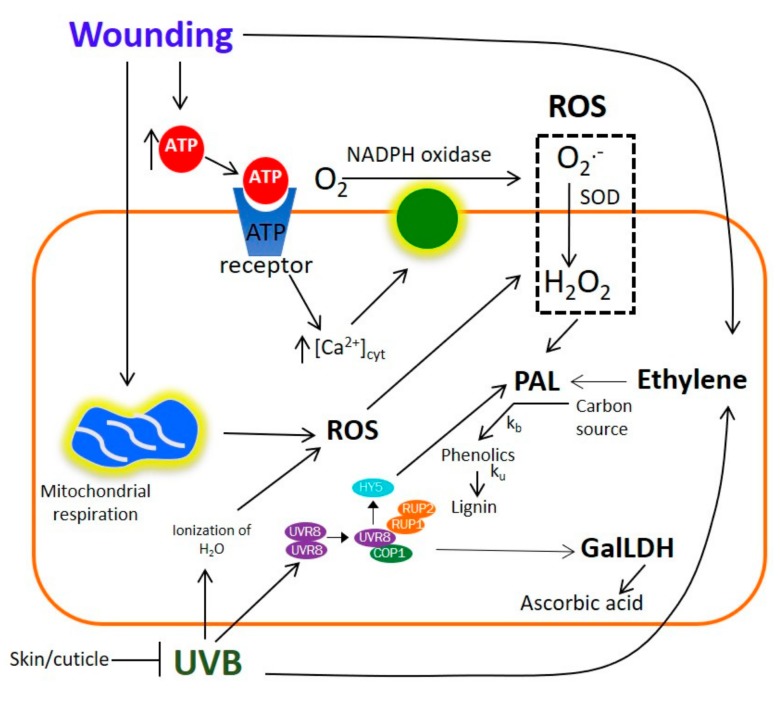
Proposed model explaining the combined effect of wounding and UVB radiation stresses on the accumulation of phenolic compounds and ascorbic acid in prickly pear. Abbreviations: L-galactono-γ-lactone dehydrogenase (GalLDH); UV RESISTANCE LOCUS 8 photoreceptor (UVR8); CONSTITUTIVELY PHOTOMORPHOGENIC 1 (COP1); REPRESSOR OF UV-B PHOTOMORPHOGENESIS 1 and 2 (RUP1/RUP2); transcription factor ELONGATED HYPOCOTYL5 (HY5); reactive oxygen species (ROS); phenylalanine ammonia-lyase (PAL).

The results obtained herein, along with previous reports, allowed for the generation of a proposed model explaining the combined effect of wounding and UVB radiation stresses on the accumulation of phenolic compounds and ascorbic acid in prickly pear (Figure 6).

In the proposed model, UVB radiation is sensed through the UVB8 photoreceptor, which activates signaling through regulators such as REPRESSOR OF UV-B PHOTOMORPHOGENESIS 1 (RUP1), CONSTITUTIVELY PHOTOMORPHOGENIC 1 (COP1), and HY5 [38,51]. Likewise, UVB produces reactive oxygen species (ROS) through partial ionization of H_2_O [28]. The skin/cuticle of plants contain UV light-filtering compounds, thus the effect by UV would be tissue-dependent. When wounding is applied prior to UV radiation, the skin/cuticle would be partially removed, and thus the underlying tissue is exposed, facilitating UV penetration which increases ROS- and UVB-induced transcription factors. Results obtained herein also suggest that the immediate activation of GalDH observed after UVB radiation and the subsequent accumulation of vitamin C as an early and late response of the stress are mediated through UVB-induced transcriptional regulators (RUP1, COP1, and HYB). The accumulation of vitamin C is further increased when UVB is applied in the wounded tissue, likely due to higher accumulation of the transcriptional regulators. For the accumulation of phenolic compounds in the wounded tissue, it has been reported that extracellular ATP released from the wounded tissue acts as the primary signal for ROS production, which acts like a secondary signal that increases the levels of PAL and phenolic compounds. Furthermore, wounding increases mitochondrial respiration (generating more ROS) and ethylene production, the latter also acting as signaling molecules for the accumulation of phenolic compounds. The accumulation of phenolic compounds observed in prickly pear is the result of a higher biosynthesis rate (Kb) than utilization rate (Ku), for lignin biosynthesis [37].

## 3. Materials and Methods

### 3.1. Chemicals

Ferric chloride (FeCl_3_), hydrochloric acid (HCl), glacial acetic acid, formic acid (CH_2_O_2_), ethyl acetate, and phosphoric acid (H_3_PO_4_) were obtained from Desarrollo de Especialidades Químicas (San Nicolás de los Garza, NL, México). The other chemicals were obtained from Sigma-Aldrich Co. (St. Louis, MO, USA).

### 3.2. Plant Material

Red prickly pears (*Opuntia ficus-indica* cv. Rojo Vigor) were obtained from the agroproducers La Flor de Villanueva, Tuna y Nopal (Puebla, Mexico), during the third week of July 2017. After harvesting, fruits were classified by maturation stage, with the size of the fruit, the external changes of color from green to red, the total soluble solids (TSS), the total titratable acids (TTA), the firmness, and the pH of the fruit all considered as indicators. Using these parameters, minimum stage of development was selected on the basis of the University of California Davis Postharvest Technology Center database [52]. The selected fruit to carry out the experiments presented the following characteristics: external color was red in the middle and green at the ends; a fresh weight of 117.88 ± 2.78 g; polar and equatorial lengths of 14.38 ± 0.69 cm and 10.28 ± 0.23 cm, respectively; total soluble solids achieved over 13.1 ± 0.10 Brix; total titratable acidity was 0.02% of citric acid equivalents; firmness of 24.41 ± 1.04 N; and pH was 6.60 ± 0.60. The methodology followed for phytochemical analyses was performed using five different fruits using the methodology previously described by Ortega-Hernández et al. [26].

### 3.3. UVB Treatments and Storage Study

UVB treatments were applied as previously described by Moreira-Rodríguez et al. [18,19] and Ortega-Hernández et al. [26]. UVB light chambers consisted of a steel framework of 60 cm (width) × 30 (depth) × 60 cm (height) covered with aluminum and equipped with four 40 W UVB lamps—Philips TL 40W/12RS (Philips, USA). The irradiation dose was determined prior to the experiment as 6.4 W·m^−2^ using a PMA 2106 UVB sensor (Solar Light, Glenside, PA, USA) measuring in the spectral range from 280–320 nm. Fruits were placed 45 cm below the light source. To ensure uniform UVB dose, fruits were aligned in rows parallel to the lamp tubes and inverted at the middle of the radiation process to achieve optimum exposure on all sides of the tissue.

A total of 20 whole and 12 wounded red prickly pears, cut in 4 pieces with a cross and a longitudinal section, using a commercial straight-edged knife, were selected for each treatment. Prior to wounding and the application of UVB radiation, the tissue was disinfected with chlorinated water (200 ppm, pH 6.5). After that, the wounded or whole tissues were treated with UVB light for 0 or 15 min. Samples were stored in darkness for 24 h in an incubator (VWR, Radnor, PA, USA) at 16 °C and 70% of relative humidity.

The immediate effect of the treatments was determined on variables such as ascorbic acid content and phenolic profiles, as well as on the enzymatic activity of phenylalanine ammonia-lyase (PAL) and L-galactono-γ-lactone dehydrogenase (GalLDH). Sampling to determine enzymatic activities was carried out at 0 and 12 h of storage, whereas ascorbic acid and phenolic profiles were determined at 0 and 24 h of storage.

Due to the amount of tissue required for the analysis of metabolites and enzyme activities, it was necessary to pool samples. The samples were obtained from five whole fruits and one piece from each wounded fruit at 0, 12, and 24 h of storage time. The peel and pulp tissues were separated (30 g), cut in small pieces, kept in polyethylene tubes, flash-frozen in liquid nitrogen, and placed at −80 °C until freeze-dried (Labconco, Kansas City, MO, USA), and then ground to a fine powder. Samples were stored at −80 °C until further analysis of phytochemicals.

### 3.4. Phytochemical Analyses

#### 3.4.1. Extraction of Phenolic Compounds

For the extraction of phenolic compounds, freeze-dried prickly pear (1 g) was homogenized with methanol (50%, 5 mL), vortexed, and sonicated for 4 min. Then, mixtures were centrifuged at 12,500 *× g* for 10 min to recover the supernatant. The pellet was re-suspended in methanol (50%, 3 mL), vortexed, sonicated for 4 min, and centrifuged at 12,500 *× g* for 10 min to recover the supernatant. Then, the pellet was re-suspended in methanol (100%, 3 mL), vortexed, sonicated for 4 min, and centrifuged under the same conditions to recover the supernatant. The supernatants obtained from the first and second extraction were combined and concentrated at 35 °C using a vacuum evaporator (EZ-2.3, Genevac Ltd., Ipswich, EN, USA). Finally, the concentrate was dissolved in methanol (100%, 2 mL) and analyzed by HPLC-DAD-MS*^n^* for the identification and quantification of phenolic compounds.

#### 3.4.2. Identification and Quantification of Phenolic Compounds

Phenolic compounds in sample extracts were quantified on a HPLC system composed of a quaternary pump, an autosampler, and a diode array detector (DAD, 1260 Infinity, Agilent Technologies, Santa Clara, CA, USA) using a 4.6 × 250 mm, 5µm C18 reverse phase column (Luna, Phenomenex, Torrance, CA, USA). Separation of phenolic compounds in the HPLC-DAD system was achieved using water (phase A) and methanol—water (60:40, *v*/*v*, phase B) adjusted to pH 2.4 with orthophosphoric acid as mobile phases. The gradient solvent system was 0/100, 3/70, 8/50, 35/30, 40/20, 45/0, 50/0, and 60/100 (min/% phase A) at a flow rate of 0.8 mL/min with an injection volume of 10 μL. Chromatographs were recorded at 280, 320, and 360 nm. Chromatographic data was processed with OpenLAB CDS ChemStation software (Agilent Technologies, Santa Clara, CA, USA).

Mass spectra of compounds were obtained on a MS Finningan LCQ Deca XP Max MSn system with a Z-spray ESI source run by Xcalibur software, version 1.3 (Thermo Finnigan-Surveyor, San Jose, CA, USA). Separations were conducted using a 4.6 × 250 mm, 5 µm C18 reverse phase column (Atlantis, Waters, Ireland). The mobile phase flow rate for phenolic compounds separation was set at 0.25 mL/min, whereas the elution gradients were performed with solvent A, water (phase A), and methanol/water (60:40, *v*/*v*, phase B) both adjusted at pH 2.4 with 5% formic acid. The gradient solvent system was 0/100, 3/70, 8/50, 35/30, 40/20, 45/0, 50/0, and 60/100 (min/% phase A). The chromatograms were monitored from 280 to 370 nm, and the complete spectral data were recorded in the range 200–600 nm. ESI was performed in the negative ionization mode, nitrogen was used as sheath gas with a flow of 50 arbitrary units, and He gas was used as dampening gas. The capillary voltage, −4.17 V; spray voltage, 5 kV; capillary temperature, 280 °C; and tube lens voltage at −55 V. Collision energies of 30% were used for the MS^n^ analysis.

The identification of individual phenolic compounds was based on their retention time, DAD spectra, and their mass-to-charge (*m*/*z*) ratio as compared with authentic standards and previous literature data [53]. For the quantification of phenolic acids, standard curves of ferulic acid (0.2–20 ppm), *p*-coumaric acid (0–75 ppm), protocatechuic acid (0–200 ppm), sinapic acid (0–15 ppm), quercetin (0–12 ppm), kaempferol (0–12 ppm), gallic acid (0–15 ppm), vanillic acid (1–25 ppm), isorhamnetin (0–25 ppm), catechin (0–25 ppm), *p*-hydroxybenzoic acid (0–25 ppm), and rosmarinic acid (0-25 ppm) were prepared. Results were expressed as mg of each individual compound per 100 g of prickly pear DW.

#### 3.4.3. Determination of Ascorbic Acid

Ascorbic acid analyses were determined using the method reported by Gillespie and Ainsworth [54]. Extractions were performed at room temperature and under dark conditions. Prickly pear sample (1 g fresh weight) was homogenized with trichloroacetic acid (TCA, 6%, 5 mL) and centrifuged at 13,000 *× g* for 20 min at 4 °C. For total ascorbic acid determination, the extract (100 µL) was mixed with dichlorodiphenyltrichloroethane (DDT) solution (32 mM, 50 µL) and incubated for 10 min at room temperature in the dark. Then, *N*-ethylmaleimide (NEM) solution (12 mM, 50 μL) was added to the mixture and incubated for 30 s. On the other hand, reduced ascorbic acid was determined adding distilled water (100 μL). Subsequently, a mixture of TCA (10%, 250 μL), H_3_PO_4_ (43%, 200 μL), α, α’-bipyridyl (4%, 200 μL), and FeCl_3_ (3%, 100 μL) solutions was added to both reactions (total and reduced). After incubation at 37 °C for 60 min, the reactions (200 μL) were placed in a 96-well microplate and absorbance readings were recorded at 525 nm. Absorbance values were compared against an ascorbic acid standard curve (0–3 mM) prepared in TCA (6%). Results were expressed as mg of ascorbic acid per 100 g of prickly pear DW.

### 3.5. Enzymatic Analyses

#### 3.5.1. Phenylalanine Ammonia-Lyase (PAL) Assay

PAL activity was measured as the conversion of L-phenylalanine to *trans-*cinnamic acid. For PAL extraction, prickly pear sample (4 g fresh weight) was added with polyvinylpyrrolidone (PVP, 0.4 g) and homogenized for 30 s with borate buffer (50 mM, pH 8.5, 16 mL), containing 2-mercaptoethanol (5 mM). The homogenates were filtered through four layers of cheesecloth, followed by centrifugation at 18,000 *× g* for 20 min at 4 °C. The supernatant was used as PAL enzyme extract. To measure PAL activity, the reaction mixture consisted on enzyme extract (80 µL) mixed with borate buffer (235 µL), and L-phenylalanine (100 mM, 35 µL) or water (blank) in a microcentrifuge tube. The reaction was incubated at 37 °C for 1 h and then stopped by adding glacial acetic acid (35 µL). Afterwards, ethyl acetate (750 µL) was added and mixed thoroughly to extract the reaction product (trans-cinnamic acid). Thereafter, the upper phase (500 µL) was recovered in a new microcentrifuge tube, dried in a vacuum evaporator (EZ-2.3, Genevac Ltd., Ipswich, EN, USA), and re-dissolved in methanol (50%, 100 µL).

The *trans-*cinnamic acid content re-dissolved in methanol was quantified by HPLC-DAD (1260 Infinity, Agilent Technologies, Santa Clara, CA, USA) using a 4.6 × 250 mm, 5µm C18 reverse phase column (Luna, Phenomenex, Torrance, CA, USA). The mobile phases consisted of water (phase A) and methanol—acetonitrile (60:40, *v*/*v*, phase B) adjusted to pH 4.5 with formic acid. The gradient solvent system was 0/100, 3/90, 10–15/75, 16/70, 25/25, 30/10, 32–34/0, and 35–40/100 (min/% phase A) at a constant flow rate of 0.6 mL/min. The identification of *trans*-cinnamic acid was on the basis of its retention time and DAD spectra characteristic as compared with an authentic standard. Chromatographs were recorded at 280 nm. The quantification of *trans*-cinnamic acid was prepared using a standard curve prepared at a range from 0.1 to 25 ppm. PAL activity was expressed as the ng of trans-cinnamic acid produced per mg of protein per hour.

#### 3.5.2. l-Galactono-γ-lactone Dehydrogenase (GalLDH) Activity

l-Galactono-γ-lactone dehydrogenase (GalLDH) activity was measured as the oxidation of L-galactono-y-lactone to ascorbic acid. Enzyme extracts were obtained according to the method of Oba, Fukui, Imai, Iriyama, and Nogami [55]. The mitochondria were extracted from prickly pear sample (3 g fresh weight) suspended in ice-cold extractive solution (3 mL) composed of Tris-HCl buffer (100 mM, pH 7.4), sucrose (400 mM), PVP (2%), and β-mercaptoethanol (50 mM) by homogenizing the mixture for 2 min. The final solution was filtered and centrifuged at 2000 *× g* for 10 min at 4 °C. The resulting supernatant was collected and centrifuged at 10,000 *× g* for 10 min at 4 °C. The precipitate was resuspended in Tris-HCl buffer (100 mM, pH 7.4, 2 mL) containing sucrose (400 mM) and centrifuged again at 10,000 *× g* for 10 min at 4 °C. This washing step was performed twice, and the final precipitate resulted in the mitochondrial fraction.

The GalLDH activity assay was performed with a protein extract prepared from the mitochondrial fraction, which was resuspended in a Tris-HCl buffer (100 mM, pH 7.4, 300 µL), containing sucrose (400 mM), ethylenediaminetetraacetic acid (EDTA, 1 mM), and Triton X-100 (1%, *v*/*v*), and then incubated for 2 h at 4 °C. The supernatant was separated and used as an extract of mitochondrial proteins for analysis of enzymatic activity. To measure the activity of GalLDH, mitochondrial protein extract (20 μL) and cytochrome c (80 mM, 100 µL) were mixed and incubated for 10 min at room temperature (25 °C). Thereafter, the enzyme substrate l-galactone-1,4-lactone (l-Gal, 4.2 mM, 100 µL) was added, and the increase in absorbance at 550 nm caused by reduction of cytochrome c (ε = 21.3 mM^−1^ cm^−1^) was monitored every 1 min for 30 min. One unit of GalLDH activity was defined as the amount of enzyme required to oxidize 1 ηmol of l-Gal (equivalent to the formation of 2 ηmol of reduced cytochrome c) per minute and expressed as oxidized ηmol of l-Gal per mg of protein per min.

### 3.6. Protein Assay

Protein content was determined from crude enzyme extracts using the Bradford method [56]. The protein extract (20 μL) was added to Bradford reagent (200 μL) in a 96-well microplate; the solution was incubated for 5 min at room temperature and the absorbance of the reaction mixture was measured at 595 nm using a microplate reader (Epoch Microplate Spectrophotometer, BioTek, Inc., Winooski, VT). A standard curve of bovine serum albumin (BSA) was prepared in the range of 0.1 to 1 mg/mL [57].

### 3.7. Respiration Rate and Ethylene Analyses

For the determination of respiration rate and VOCs (related with ethylene production) three whole fruits and five pieces obtained from five independent cut red prickly pears of each treatment were incubated in sealed plastic jars (1.9 L) at room temperature (22 °C) and allowed to accumulate CO_2_ and VOCs for 1 h. Gas samples (15 mL) were obtained using a needle probe connected to the intake of the F-950 gas analyzer (Felix Instruments, WA, USA) as previously reported by Cuellar-Villarreal et al. [58].

### 3.8. Statistical Analysis

Statistical analyses of chemical analyses were performed using three treatment repetitions unless otherwise indicated in the figures. Data represent the mean values of samples and their standard error. A full-factorial design was applied to study the main effects and interactions of the variables evaluated. Analyses of variance (ANOVA) were conducted using JMP software version 13.0 (SAS Institute Inc., Cary, NC, USA) and mean separations were performed using the least significant difference (LSD) test (*p* < 0.05).

## 4. Conclusions

In the present study, results showed that the application of UVB light and wounding stress can induce the biosynthesis and accumulation of secondary metabolites in red prickly pear. The treatment of UVB alone during 15 min to whole fruit was the most effective treatment to induce the biosynthesis of phenolic compounds in pulp and peel after 24 h of storage. On the other hand, the combination of UVB for 15 min and wounding stress had a synergistic effect on the accumulation of ascorbic acid after 24 h of storage due to exposure of the underlying tissue when the peel is removed. Respiration rate and ethylene production increased due to the application of stresses, suggesting that ROS and ethylene are acting as signaling molecules modulating the stress response of phenolic biosynthesis. The stressed tissue could be used as raw material for the production of functional foods or for the extraction and purification of compounds with applications in the pharmaceutical and dietary supplements industries. Further research should be directed into performing physiological and molecular biology experiments to obtain a deeper understanding on the molecular mechanism involved in the stress-response reported in the proposed model herein.

## Figures and Tables

**Table:** (**a**)

Treatment		Storage Time	Phenolic Compounds Concentration (mg/100 g) DW ^1,2,3,4^						
			Catechin	*p*-Hydroxybenzoic acid	Protocatechuic acid	Gallocatechin	Kaempferol	Sinapoyl malate	1-*O*-β-d-glucopyranosyl sinapate
**Control**		0 h	11.54 ± 1.26 ^a^	42.14 ± 0.95 ^ab^	197.08 ± 14.51 ^cd^	15.79 ± 1.08 ^ab^	23.49 ± 2.00 ^c^	26.89 ± 1.12 ^de^	1.17 ± 0.08 ^d^
UV-	Whole	24 h	11.99 ± 1.39 ^a^	44.27 ± 3.61 ^a^	212.86 ± 14.41 ^c^	17.22 ± 1.12 ^a^	20.71 ± 2.09 ^c^	24.36 ± 0.93 ^e^	1.20 ± 0.03 ^d^
	Wounded		8.44 ± 1.25 ^c^	38.94 ± 2.17 ^b^	220.73 ± 15.02 ^c^	14.13 ± 0.89 ^b^	21.17 ± 2.14 ^c^	29.47 ± 1.26 ^d^	1.36 ± 0.17 ^d^
UV+	Whole	0 h	10.20 ± 0.23 ^b^	39.40 ± 0.84 ^b^	259.90 ± 18.12 ^b^	13.09 ± 0.15 ^c^	29.85 ± 2.83 ^b^	31.55 ± 1.80 ^d^	1.63 ± 0.03 ^c^
	Wounded		8.15 ± 0.21 ^c^	46.35 ± 0.64 ^a^	230.85 ± 14.31 ^b^	11.66 ± 0.78 ^c^	31.43 ± 2.05 ^b^	42.83 ± 1.09 ^c^	1.86 ± 0.07 ^b^
	Whole	24 h	12.29 ± 0.12 ^a^	39.93 ± 0.45 ^ab^	299.94 ± 17.84 ^a^	17.82 ± 1.23 ^a^	34.07 ± 3.79 ^b^	70.10 ± 2.32 ^b^	1.90 ± 0.03 ^b^
	Wounded		6.09 ± 0.86 ^d^	37.49 ± 0.65 ^b^	215.21 ± 7.67 ^c^	15.71 ± 1.73 ^ab^	62.92 ± 4.87 ^a^	93.56 ± 6.42 ^a^	7.87 ± 2.39 ^a^
**Significance**									
UVB			NS	***	***	***	***	***	***
Wounding (W)			***	***	***	***	***	***	***
Storage time (ST)			***	***	***	***	***	***	***
UVB × W			**	*	***	***	***	***	***
UVB × ST			**	NS	***	***	***	***	***
W × ST			***	***	***	***	***	***	***
UVB × W × ST			**	NS	**	***	***	***	***

^1^ Compounds were quantified at 280 nm: catechin, p-hydroxybenzoic acid, protocatechuic acid, gallocatechin; 320 nm: sinapoyl malate, 1-*O*-β-d-glucopyranosyl sinapate ^2^ Values represent the mean of three replicates ± standard error of the mean. ^3^ Different letters in the same column indicate statistical difference in the concentration of each compound between treatments using the least significant difference (LSD) test (*p* < 0.05). ^4^ Asterisks indicate that main effects and interactions are significantly different by analyses of variance (ANOVA). NS—non significant, * *p* < 0.05, ** *p* < 0.01, *** *p* < 0.001. Abbreviations: dry weight (DW); fruit without UVB light treatment (UV-); fruit treated with UVB (6.4 W·m^−2^) light for 15 min (UV+); ND (not detected).

**Table:** (**b**)

Treatment		Storage Time	Phenolic Compounds Concentration (mg/100 g) DW ^1,2,3,4^						
			Rosmarinic acid	Vanillic acid	Gallic acid	*p*-Coumaric acid	Sinapic acid	Ferulic acid	Caffeic acid derivative
**Control**		0 h	5.83 ± 0.21 ^c^	6.87 ± 0.85 ^c^	15.74 ± 0.54 ^ab^	1.53 ± 0.15 ^c^	0.96 ± 0.04 ^cd^	1.42 ± 0.07 ^c^	1.19 ± 0.04 ^c^
UV-	Whole Wounded	24 h	4.50 ± 0.27 ^d^	6.17 ± 0.62 ^c^	14.85 ± 1.45 ^b^	1.36 ± 0.37 ^c^	1.04 ± 0.15 ^cd^	1.32 ± 0.18 ^c^	1.29 ± 0.15 ^c^
			6.56 ± 0.79 ^c^	5.69 ± 2.39 ^d^	13.93 ± 1.62 ^b^	1.93 ± 0.36 ^b^	1.48 ± 0.03 ^b^	1.72 ± 0.28 ^b^	1.64 ± 0.05 ^b^
UV+	Whole	0 h	5.58 ± 1.12 ^c^	6.47 ± 0.08 ^c^	17.04 ± 0.10 ^a^	1.69 ± 0.10 ^bc^	1.06 ± 0.06 ^cd^	1.31 ± 0.03 ^cd^	1.27 ± 0.02 ^c^
	Wounded		8.65 ± 0.40 ^b^	7.86 ± 0.16 ^a^	16.64 ± 2.26 ^a^	8.25 ± 0.05 ^a^	2.99 ± 0.06 ^a^	6.99 ± 0.07 ^a^	5.65 ± 0.01 ^a^
	Whole	24 h	30.80 ± 2.35 ^a^	7.09 ± 0.27 ^ab^	13.49 ± 0.31 ^b^	0.64 ± 0.19 ^e^	1.28 ± 0.04 ^b^	1.34 ± 0.02 ^cd^	1.45 ± 0.01 ^c^
	Wounded		26.87 ± 3.77 ^a^	5.14 ± 0.03 ^cd^	12.70 ± 0.06 ^b^	0.93 ± 0.06 ^d^	1.16 ± 0.14 ^c^	1.28 ± 0.11 ^d^	1.18 ± 0.09 ^c^
**Significance**									
UVB			***	**	***	NS	***	NS	NS
Wounding (W)			***	*	***	***	***	***	***
Storage time (ST)			***	NS	***	NS	***	NS	*
UVB × W			***	*	**	**	***	***	***
UVB × ST			***	*	**	***	***	**	***
W × ST			***	NS	NS	NS	***	NS	***
UVB × W × ST			***	*	*	***	***	***	***

^1^ Compounds were quantified at 320 nm. ^2^ Values represent the mean of three replicates ± standard error of the mean. ^3^ Different letters in the same column indicate statistical difference in the concentration of each compound between treatments using the least significant difference (LSD) test (*p* < 0.05). ^4^ Asterisks indicate that main effects and interactions are significantly different by analyses of variance (ANOVA). NS—non significant, * *p* < 0.05, ** *p* < 0.01, *** *p* < 0.001. Abbreviations: dry weight (DW); fruit without UVB light treatment (UV-); fruit treated with UVB (6.4 W·m^−2^) light for 15 min (UV+); ND (not detected).

**Table:** (**c**)

Treatment		Storage Time	Phenolic Compounds Concentration (mg/100 g) DW ^1,2,3,4^						
			Rutin	Quercetin 3-*O*-glucoside	Kaempferol 3-*O*-glucoside	Quercetin	Kaempferol 3-*O*-sophoroside-7*O*-glucoside	Isorhamnetin	Total
**Control**		0 h	1.08 ± 0.06 ^b^	0.91 ± 0.04 ^d^	0.38 ± 0.02 ^d^	33.18 ± 2.30 ^c^	0.72 ± 0.11 ^c^	1.80 ± 0.03 ^ab^	389.70 ± 32.15 ^e^
UV-	Whole	24 h	1.25 ± 0.11 ^a^	0.97 ± 0.06 ^d^	0.35 ± 0.00 ^d^	36.20 ± 1.03 ^c^	0.63 ± 0.06 ^d^	1.66 ± 0.15 ^ab^	404.18 ± 27.09 ^e^
	Wounded		1.07 ± 0.01 ^b^	2.23 ± 0.00 ^c^	0.49 ± 0.04 ^c^	36.28 ± 1.12 ^c^	0.69 ± 0.05 ^c^	1.71 ± 0.13 ^ab^	409.67 ± 16.56 ^e^
UV+	Whole	0 h	1.24 ± 0.00 ^a^	0.81 ± 0.02 ^d^	0.60 ± 0.02 ^b^	33.28 ± 2.01 ^c^	1.05 ± 0.07 ^b^	1.51 ± 0.04 ^ab^	458.52 ± 32.42 ^de^
	Wounded		1.13 ± 0.00 ^ab^	4.23 ± 0.00 ^b^	1.05 ± 0.00 ^b^	47.53 ± 1.30 ^a^	1.35 ± 0.00 ^a^	1.69 ± 0.03 ^ab^	487.25 ± 28.78 ^c^
	Whole	24 h	1.24 ± 0.01 ^a^	1.24 ± 0.00 ^c^	0.05 ± 0.00 ^e^	44.14 ± 3.00 ^b^	0.99 ± 0.03 ^b^	1.54 ± 0.00 ^ab^	581.34 ± 43.36 ^a^
	Wounded		0.97 ± 0.00 ^bc^	7.79 ± 0.38 ^a^	6.82 ± 0.45 ^a^	52.13 ± 3.94 ^a^	1.49 ± 0.18 ^a^	1.90 ± 0.04 ^a^	559.21 ± 37.85 ^b^
**Significance**									
UVB			***	***	***	***	***	***	***
Wounding (W)			***	***	***	***	*	***	***
Storage time (ST)			***	***	***	***	**	***	***
UVB × W			***	***	***	***	**	***	***
UVB × ST			***	***	***	***	***	***	***
W × ST			***	***	***	***	***	***	***
UVB × W × ST			***	***	***	***	***	***	***

^1^ Compounds were quantified at 360 nm. ^2^ Values represent the mean of three replicates ± standard error of the mean. ^3^ Different letters in the same column indicate statistical difference in the concentration of each compound between treatments using the least significant difference (LSD) test (*p* < 0.05). ^4^ Asterisks indicate that main effects and interactions are significantly different by analyses of variance (ANOVA). NS—non significant, * *p* < 0.05, ** *p* < 0.01, *** *p* < 0.001. Abbreviations: dry weight (DW); fruit without UVB light treatment (UV-); fruit treated with UVB (6.4 W·m^−2^) light for 15 min (UV+); ND (not detected).

**Table:** (**a**)

Treatment		Storage Time	Phenolic Compounds Concentration (mg/100 g) DW ^1,2,3,4^						
			Catechin	p-Hydroxybenzoic acid	Protocatechuic acid	Gallocatechin	Kaempferol	Sinapoyl malate	1-*O*-β-d-Glucopyranosyl sinapate
**Control**		0 h	46.55 ± 3.60 ^b^	13.67 ± 0.95 ^a^	453.05 ± 31.56 ^a^	29.91 ± 1.93 ^a^	6.01 ± 1.39 ^e^	55.48 ± 2.28 ^f^	10.00 ± 0.79 ^e^
UV-	Whole	24 h	42.72 ± 2.55 ^b^	11.21 ± 0.36 ^b^	390.78 ± 24.24 ^b^	26.52 ± 1.03 ^ab^	5.60± 4.38 ^f^	75.04 ± 4.63 ^e^	25.36 ± 0.88 ^d^
	Wounded		51.20 ± 3.67 ^ab^	12.98 ± 1.03 ^a^	468.71 ± 41.41 ^a^	33.47 ± 2.72 ^a^	6.20 ± 2.41 ^e^	58.30 ± 2.49 ^f^	8.13 ± 1.67 ^e^
UV+	Whole	0 h	43.20 ± 2.45 ^b^	7.33 ± 0.14 ^c^	216.37 ± 19.12 ^d^	24.53 ± 2.51 ^b^	38.14 ± 2.17 ^a^	86.92 ± 2.37 ^d^	38.10 ± 1.51 ^cd^
	Wounded		37.78 ± 2.32 ^c^	6.47 ± 0.78 ^cd^	389.04 ± 28.30 ^b^	21.67 ± 1.82 ^c^	28.66 ± 1.29 ^c^	128.99 ± 4.95 ^c^	43.47 ± 1.55 ^bc^
	Whole	24 h	45.12 ± 2.86 ^b^	4.00 ± 0.78 ^d^	412.00 ± 37.55 ^ab^	18.84 ± 2.35 ^d^	32.60 ± 1.44 ^b^	158.83 ± 12.65 ^b^	49.21 ± 0.82 ^b^
	Wounded		32.25 ± 2.82 ^d^	2.63 ± 0.89 ^e^	330.77 ± 29.04 ^c^	12.04 ± 1.78 ^e^	23.31 ± 2.41 ^d^	202.26 ± 18.81 ^a^	55.46 ± 5.20 ^a^
**Significance**									
UVB			***	NS	***	***	***	***	***
Wounding (W)			NS	***	***	***	***	***	***
Storage time (ST)			NS	***	***	NS	NS	***	***
UVB × W			NS	***	*	***	***	NS	NS
UVB × ST			***	***	NS	***	***	NS	NS
W × ST			***	***	NS	***	***	*	***
UVB × W × ST			***	***	*	***	***	NS	NS

^1^ Compounds were quantified at 280 nm: catechin, p-hydroxybenzoic acid, protocatechuic acid, gallocatechin; 320 nm: sinapoyl malate, 1-*O*-β-d-glucopyranosyl sinapate. ^2^ Values represent the mean of three replicates ± standard error of the mean. ^3^ Different letters in the same column indicate statistical difference in the concentration of each compound between treatments using the least significant difference (LSD) test (*p* < 0.05). ^4^ Asterisks indicate that main effects and interactions are significantly different by analyses of variance (ANOVA). NS—non significant, * *p* < 0.05, ** *p* < 0.01, *** *p* < 0.001. Abbreviations: dry weight (DW); fruit without UVB light treatment (UV-); fruit treated with UVB (6.4 W·m^−2^) light for 15 min (UV+); ND (not detected).

**Table:** (**b**)

Treatment		Storage Time	Phenolic Compounds Concentration (mg/100 g) DW ^1,2,3,4^						
			Rosmarinic acid	Vanillic acid	Gallic acid	*p*-Coumaric acid	Sinapic acid	Ferulic acid	Caffeic acid derivative
**Control**		0 h	38.73 ± 1.75 ^e^	8.15 ± 0.69 ^a^	7.27 ± 0.88 ^ab^	1.32 ± 0.15 ^d^	2.33 ± 0.09 ^d^	1.55 ± 0.14 ^d^	0.74 ± 0.06 ^c^
UV-	Whole	24 h	54.56 ± 2.57 ^d^	7.13 ± 1.00 ^ab^	8.13 ± 0.15 ^a^	1.82 ± 0.04 ^c^	4.47 ± 0.58 ^c^	2.92 ± 0.35 ^c^	2.70 ± 0.09 ^a^
	Wounded		42.02 ± 5.79 ^de^	8.53 ± 0.51 ^a^	6.51 ± 0.03 ^b^	1.23 ± 0.45 ^d^	2.11 ± 0.18 ^d^	1.39 ± 0.61 ^d^	0.61 ± 0.29 ^d^
UV+	Whole	0 h	87.11 ± 6.86 ^c^	7.10 ± 1.38 ^b^	7.13 ± 0.10 ^ab^	1.91 ± 0.12 ^c^	5.76 ± 0.08 ^c^	1.71 ± 0.27 ^d^	0.69 ± 0.02 ^d^
	Wounded		141.97 ± 6.06 ^b^	6.95 ± 0.04 ^b^	7.95 ± 0.03 ^a^	8.37 ± 1.12 ^a^	14.36 ± 0.93 ^b^	21.15 ± 1.01 ^a^	1.87 ± 0.05 ^b^
	Whole	24 h	209.11 ± 0.38 ^a^	3.94 ± 0.26 ^d^	7.45 ± 0.08 ^ab^	1.90 ± 0.13 ^c^	15.63 ± 1.21 ^b^	5.36 ± 1.99 ^b^	0.81 ± 0.02 ^c^
	Wounded		155.71 ± 8.55 ^b^	5.59 ± 2.05 ^c^	7.77 ± 0.72 ^a^	2.83 ± 0.69 ^b^	24.89 ± 0.77 ^a^	5.5± 0.57 ^b^	2.99 ± 0.07 ^a^
**Significance**									
UVB			NS	*	***	***	***	NS	***
Wounding (W)			***	NS	*	***	***	NS	***
Storage time (ST)			NS	NS	***	***	**	NS	***
UVB × W			NS	*	NS	***	***	NS	NS
UVB × ST			NS	NS	***	**	***	NS	***
W × ST			***	NS	NS	***	*	NS	**
UVB × W × ST			***	*	NS	***	***	NS	**

^1^ Compounds were quantified at 320 nm. ^2^ Values represent the mean of three replicates ± standard error of the mean. ^3^ Different letters in the same column indicate statistical difference in the concentration of each compound between treatments using the least significant difference (LSD) test (*p* < 0.05). ^4^ Asterisks indicate that main effects and interactions are significantly different by analyses of variance (ANOVA). NS—non significant, * *p* < 0.05, ** *p* < 0.01, *** *p* < 0.001. Abbreviations: dry weight (DW); fruit without UVB light treatment (UV-); fruit treated with UVB (6.4 W·m^−2^) light for 15 min (UV+); ND (not detected).

**Table:** (**c**)

Treatment		Storage Time	Phenolic Compounds Concentration (mg/100 g) DW ^1,2,3,4^						
			Rutin	Quercetin 3-O-glucoside	Kaempferol 3-O-glucoside	Quercetin	Kaempferol 3-O-sophoroside-7O-glucoside	Isorhamnetin	Total
**Control**		0 h	9.09 ± 0.32 ^a^	24.59 ± 0.27 ^d^	13.98 ± 0.45 ^c^	70.74 ± 3.39 ^e^	1.28 ± 0.14 ^e^	17.29 ± 0.86 ^a^	811.74 ± 48.64 ^de^
UV-	Whole	24 h	7.34 ± 0.64 ^bc^	21.26 ± 6.59 ^d^	10.22 ± 0.41 ^cd^	63.29 ± 4.38 ^ef^	1.08 ± 0.24 ^e^	14.14 ± 1.22 ^ab^	776.29 ± 260.14 ^e^
	Wounded		10.34 ± 0.14 ^a^	22.90 ± 1.05 ^d^	12.86 ± 1.48 ^c^	78.32 ± 2.41 ^e^	1.92 ± 0.31 ^d^	15.63 ± 0.73 ^ab^	843.37 ± 275.24 ^de^
UV+	Whole	0 h	8.55 ± 0.19 ^ab^	59.90 ± 5.74 ^a^	31.25 ± 1.81 ^a^	252.74 ± 24.03 ^d^	4.71 ± 0.17 ^b^	16.41 ± 1.95 ^a^	939.61 ± 89.50 ^d^
	Wounded		8.20 ± 1.42 ^ab^	43.86 ± 3.59 ^c^	19.34 ± 3.48 ^b^	312.16 ± 21.29 ^c^	2.33 ± 0.21 ^c^	14.44 ± 0.26 ^ab^	1259.38 ± 96.94 ^c^
	Whole	24 h	9.45 ± 0.32 ^a^	53.54 ± 2.03 ^b^	33.55 ± 0.17 ^a^	572.60 ± 40.33 ^a^	5.05 ± 0.00 ^a^	15.11 ± 0.58 ^ab^	1654.06 ± 118.02 ^a^
	Wounded		6.57 ± 5.70 ^c^	42.46 ± 6.30 ^c^	22.91 ± 1.96 ^b^	428.65 ± 30.41 ^b^	1.49 ± 0.37 ^d^	10.05 ± 0.76 ^b^	1376.19 ± 134.56 ^b^
**Significance**									
UVB			***	NS	***	NS	NS	NS	***
Wounding (W)			***	NS	NS	**	***	***	***
Storage time (ST)			NS	NS	***	**	**	NS	***
UVB × W			***	NS	***	NS	***	NS	*
UVB × ST			***	**	***	***	***	***	NS
W × ST			**	NS	***	NS	NS	*	NS
UVB × W × ST			***	NS	**	*	NS	***	*

^1^ Compounds were quantified at 360 nm. ^2^ Values represent the mean of three replicates ± standard error of the mean. ^3^ Different letters in the same column indicate statistical difference in the concentration of each compound between treatments using the least significant difference (LSD) test (*p* < 0.05). ^4^ Asterisks indicate that main effects and interactions are significantly different by analyses of variance (ANOVA). NS—non significant, * *p* < 0.05, ** *p* < 0.01, *** *p* < 0.001. Abbreviations: dry weight (DW); fruit without UVB light treatment (UV-); fruit treated with UVB (6.4 W·m^−2^) light for 15 min (UV+); ND (not detected).
